# The incidence of genetic disease alleles in Australian Shepherd dog breed in European countries

**DOI:** 10.1371/journal.pone.0281215

**Published:** 2023-02-27

**Authors:** Zuzana Majchrakova, Evelina Hrckova Turnova, Marcela Bielikova, Jan Turna, Andrej Dudas

**Affiliations:** 1 Slovgen Ltd., Diagnostic Laboratory, Bratislava, Slovakia; 2 Comenius University Science Park, Bratislava, Slovakia; 3 Department of Molecular Biology, Faculty of Natural Sciences, Comenius University, Bratislava, Slovakia; University of Jordan, JORDAN

## Abstract

Genetic disease control is generally not given the importance it deserves. Information about what percentage of individuals carry a disorder-causing mutation is crucial for breeders to produce healthy offspring and maintain a healthy dog population of a particular breed. This study aims to provide information about the incidence of mutant alleles for the most frequently occurring hereditary diseases in the Australian Shepherd dog breed (AS). The samples were collected during a 10-years period (2012–2022) in the European population of the AS. Mutant alleles and incidence were calculated from all the obtained data for all the diseases, specifically: collie eye anomaly (9.71%), canine multifocal retinopathy type 1 (0.53%), hereditary cataract (11.64%), progressive rod-cone degeneration (1.58%), degenerative myelopathy (11.77%) and bob-tail/short-tail (31.74%). Our data provide more information to dog breeders to support their effort to limit the spread of hereditary diseases.

## Introduction

Access to accurate genetic testing results for hereditary disorders and information about their incidence in purebred dog populations allows breeders to select the right individuals for mating to obtain healthy offspring, thus suppressing the incidence of the disease. Moreover, understanding genetic disease’s background can bring more effective treatments.

This study has focused on the incidence of wild-type and mutant alleles of six genetic disorders occurring in the Australian Shepherd breed (AS). The AS developed in western states of the US, contrary to what its name suggests. The breed is likely descended from shepherd breeds used by Basque sheepherders in the borderland region between France and Spain. Their ancestors followed the Basques to Australia, where they were crossed with other shepherd dogs like Collie and Border Collie, until they eventually found their way to the US during the Gold Rush era along with the sheep they herded. Here the breed quickly became the herding dog of choice for American cowboys, who referred to it as AS. In 1957 the Australian Shepherd Club of America (ASCA) was formed, and the breed standard was established twenty years later. The AS was admitted as a breed by the American Kennel Club in 1993 [1, 2].

Although considered a generally healthy breed, there are several hereditary disease alleles reported in AS (Table 1), namely: collie eye anomaly (CEA), canine multifocal retinopathy type 1 (CMR1), hereditary cataract (HC), multidrug sensitivity (MDR1), progressive rod-cone degeneration (PRA-PRCD), degenerative myelopathy (DM) and bob-tail/short-tail (SHT/NBT)—technically not a disease, but mating two homozygous mutants produce puppies with spinal cord defects, moreover, in a majority of cases the homozygous GG allele combination is lethal in early foetal life [3]. This article does not cover multidrug sensitivity, as we have already analysed the incidence of MDR1 mutation in affected breeds, including AS, in our previous work [4].

**Table 1:** An overview of six genetic disorders with their corresponding gene, chromosome number, genomic location, mutation, effect, inheritance, OMIA number and human homologue.

Disorder	Gene	CFA	Genomic location	Mutation	Effect	Inheritance	OMIA number	Human homologue
**Collie eye anomaly (CEA)**	*NHEJ1*	CFA37	g.25698028_25705826del	c.588+462_588+260del	XM_005640671.1; a deletion of 7799bp in the NHEJ1 gene	AR	OMIA 000218-9615	Severe combined immunodeficiency (SCID) with microcephaly, growth retardation, and sensitivity to ionizing radiation (IR)
								https://omim.org/entry/611290
**Canine multifocal retinopathy, type 1 (CMR1)**	*BEST1*	CFA18	g.54478586G>A	c.73C>T	p.(R25*)	AR	OMIA 001444-9615	Macular dystrophy
								https://omim.org/entry/153700
								Bestrophinopathy
								https://omim.org/entry/611809
								Vitreoretinochoroidopathy
								https://omim.org/entry/193220
								Retinitis pigmentosa
								https://omim.org/entry/613194
**Degenerative myelopathy (DM)**	*SOD1*	CFA31	g.26540342G>A	c.118G>A	p.(E40K)	AR	OMIA 000263-9615	Amyotrophic lateral sclerosis
								https://omim.org/entry/105400
**Primary hereditary cataract (HSF4)**	*HSF4*	CFA5	g.82198114_82198115insG	c.971_972insC	p.(P324Hfs*87)	AR	OMIA 001758-9615	Cataract
								https://omim.org/entry/116800
**Hyperuricosuria (HUU)**	*SLC2A9*	CFA3	g.69456869G>T	c.563G>T	p.(C188F)	AR	OMIA 001033-9615	Hypouricemia 1 and 2
								https://omim.org/entry/612076
								https://omim.org/entry/220150
**Malignant hyperthermia (MH)**	*RYR1*	CFA15	g.114562165A>G	c.1643T>C	p.(V548A)	AR	OMIA 000621–9615	Malignant hyperthermia
								https://omim.org/entry/145600
**Progressive rod-cone degeneration (PRCD)**	*PRCD*	CFA9	g.4188663C>T	c.5G>A	p.(C2Y)	AR	OMIA 001298-9615	Retinitis pigmentosa
								https://omim.org/entry/610599
**Short tail (SHT)**	*T—gene*	CFA1	g.54192143G>C	c.189C>G	p.(I63M)	AD	OMIA 000975-9615	-

Collie eye anomaly (CEA) is a hereditary autosomal recessive disease in sheep-herding breeds caused by abnormal embryonic development, which results in severe eye deformations (choroidal hypoplasia, coloboma, staphyloma and retinal detachment) and, in the final stages, even blindness. This disease was first noticed in 1953 in Rough Collie dogs; since then, more affected breeds have been identified [5–8]. Collie Rough, Collie Smooth, Border Collie, Shetland Sheepdog and AS appear to be in the same cluster and share a common Collie ancestor, so causative mutation causing CEA in the *NHEJ1* gene seems to be identical by descent and is one of the top three most common congenital ocular disorders that affect AS [9–11]. The deletion of 7799 bp is located in intron 4 (67 kbp) of the *NHEJ1* gene on chromosome CFA37 [5].

The bilateral focal serous detachments of the retina and multiple fundic lesions are the primary symptoms of canine multifocal retinopathy (CMR) which belongs to a group of retinal diseases called bestrophinopathies, which can also be found in humans [12, 13]. The autosomal recessive CMR type 1 disease is caused by a stop mutation in the *BEST1* gene (C73T). It affects 11 dog breeds worldwide derived from the Mastiff line and, surprisingly, not genetically related to the AS dogs [13–15]. The *BEST1* gene encodes 66 kDa transmembrane protein found in the retina (retinal pigment epithelium, choroid) and in small amounts in the brain. The transition C73T in the N-terminal domain leads to the production of a premature stop codon and the formation of a truncated anion channel protein 25 codons long instead of 580 codons in wild-type form [16].

The adult-onset canine degenerative myelopathy (DM) belongs to a family of muscular neurodegenerative disorders characterised by the progressive destruction of neuronal motor functions and demyelination of sheaths surrounding the nerve fibres. It represents a great canine disease model for superoxide dismutase 1 associated with human amyotrophic lateral sclerosis [17]. In 2009, the mutation associated with canine DM was described as a substitution of G to A in a highly conserved Cu/Zn—superoxide dismutase 1 (*SOD1*) gene on chromosome 31 (c.118G to A, p.E40K) [18]. A histological examination reveals insoluble inclusions of misfolded proteins in the cytoplasm of motor neurons in the spinal cord sections, which are also typical for human amyotrophic lateral sclerosis [19, 20].

The bilaterally symmetrical and progressive hereditary cataract (HC) typically results in complete blindness of homozygous mutants. This non—congenital disability could be generally detected between 8 to 12 weeks after birth. Lens opacity and progression leading to the first signs of blindness appear around 2 to 3 years of age [21, 22]. The mutation associated with HC was identified in the *HSF4* gene (heat shock transcription factor 4) located on chromosome 5. HC in Staffordshire Bull Terrier, Boston Terrier and French Bulldog breeds is an autosomal recessively inherited disease. All affected individuals carry a single nucleotide insertion of cytosine in exon 9 (CFA5 g85286582–85286583insC) in the *HSF4* gene. On the other hand, in the AS breed, the exact same location contains a different mutation—cytosine deletion (g.85286582delC) and inheritance was described as an autosomal dominant form with incomplete penetrance. Both mutations, insertion and deletion, lead to a frameshift mutation and create a premature stop codon, resulting in a truncated and aberrant protein and failure of its function [21–24].

The late onset autosomal recessive canine progressive rod-cone degeneration (PRCD) belongs to a large group of phenotypically very similar retinal disorders called progressive retinal atrophies [14, 25]. The first five to seven years of the postnatal development of photoreceptors seem to be with no structural and functional degeneration. However, the destruction of the outer segments of both photoreceptors can be seen, eventually leading to total bilateral blindness. Deterioration of rods was found to be faster than cones [26–28]. The ∼600 bp long *PRCD* gene encodes a ∼6 kDa S-acetylated rhodopsin-binding protein consisting of 54 amino acids. It can be especially found in photoreceptor discs, where it plays a crucial role in the photoreceptor disc morphogenesis by keeping invaginating membranes of new discs tightly close together [29, 30]. The equivalent mutation c.5G>A resulting in the C2Y substitution in dogs can also be observed in human retinitis pigmentosa (RP) due to the highly conserved region in all vertebrates. Hence dogs represent a great model for RP research which can be widely studied [29, 31].

Haworth et al. described the mutation causing short-tail phenotype in dogs for the first time in 2001 [32]. In 2009, Hytonen et al. revealed that the missense mutation responsible for this condition could be found in 17 dog breeds, including AS [33]. The substitution C to G (c.189C to G) in a canine homologue of the T-box transcription factor T is transmitted as autosomal dominant. The result is a defective protein that does not bind to its target DNA. All phenotypically homozygous mutants were found to be only heterozygotes, which leads to the conclusion that the presence of both mutant alleles (GG) is not compatible with foetal development because it is lethal [3, 32, 33]. This statement is supported by the fact that only two tailless puppies of the Welsh Corgi Pembroke from two litters were homozygous for the mutant allele, but one died a few seconds after birth [3].

These diseases cause mild to serious health issues for the affected animals. Moreover, most of the mentioned diseases have similar or identical backgrounds in humans. Thus, dogs appear to be great animal models. An overview of six genetic disorders with their corresponding gene, chromosome number, genomic location, mutation, effect, inheritance, OMIA number and similar human models are listed in Table 1.

## Materials and methods

### Ethical statement

The research was conducted in full compliance and strict accordance with the ethical codex of Comenius University in Bratislava.

### DNA samples and isolation

DNA samples of AS were submitted by dog owners or their veterinarians. In most cases, they provided us with the chip number, the dog’s full name, the kennel’s name, and the studbook entry number. Buccal samples were collected using a cytological brush or swab, and EDTA blood samples were obtained from veterinarians. DNA was extracted using Gentra Puregene Blood Kit (Qiagen), QIAamp DNA Investigator Kit (Qiagen) or DNeasy Blood & Tissue Kit (Qiagen) according to the manufacturer’s instructions. All samples were stored at 4°C until further analysis. In cooperation with Slovgen diagnostic laboratory, a total number of 2595 dogs were analysed during the period of 10 years (CEA—1503 samples, CMR1–376 samples, DM—722 samples, HC—1641 samples, PRA-PRCD—1454 samples and SHT—282 samples).

### Mutation analysis and genotyping

The required part of genomic DNA was amplified using primers listed in S1 Table to determine the presence or the absence of the mutant allele for each analysed gene.

PCR mixture for each amplification reaction contained 2–2.5 mM MgCl_2_, 1x Dream Taq buffer (Thermo Fisher Scientific), 1 μM^1^ of both forward and reverse primer, 0.25 mM dNTPs (Thermo Fisher Scientific), 1 U Dream Taq polymerase (Thermo Fisher Scientific), approximately 50 ng of template DNA and H_2_O.

We used PCR followed by the restriction fragment length polymorphism (RFLP) analysis to detect alleles of CMR1, DM, PRCD and SHT alleles. The restriction enzyme digestion was performed in a 20 μl reaction mixture which consisted of 2 U of the restriction endonuclease (*HphI*—CMR1, *Eco57I* - DM, *SfaNI*—HUU, *AlwI* and *RsaI*—PRCD, *FD-Eco91I* - SHT) (Thermo Fisher Scientific), 1x supplied buffer, 10 μl PCR product and distilled water. Fragments were separated by size using electrophoresis on 1.5% agarose gel or 10% polyacrylamide gel, depending on the product length.

Sequencing was used for HSF4 mutation screening and for verifying the results of RFLP methods. The five samples were chosen randomly from each genotype available, and the sequencing data were compared with the results of the RFLP analysis.

The PCR products were directly sequenced after ExoSAP-IT (Applied Biosystems) treatment using PCR primers with the ABI BigDye Terminator Sequencing Kit 3.1 (Applied Biosystems) on an ABI 3500 capillary sequencer. Sequence data were analysed with Vector NTI Advance 7.0 (Invitrogen).

## Results

Over a period of 10 years, together 2595 samples from privately owned pet dogs from the European population of AS were collected and analysed, namely: CEA—1503 pcs, CMR1–376 pcs, DM—722 pcs, HC—1641 pcs, PRA-PRCD—1454 pcs and SHT 282 pcs. These data were obtained in cooperation with the Slovgen diagnostic laboratory (Bratislava, Slovakia), where tests were performed as a part of its diagnostic services. Health status, medical records, treatments, and other documentation of tested dogs were unavailable due to ownership mostly by private breeders and owners. They have provided us with basic dog identifiers like breed, gender, chip number and studbook entry number. Blood relations among analysed individuals were not known. Therefore, it can be assumed that the Hardy-Weinberg principle does not apply considering breeding methods (for example selection, non-random mating).

**Fig 1:**
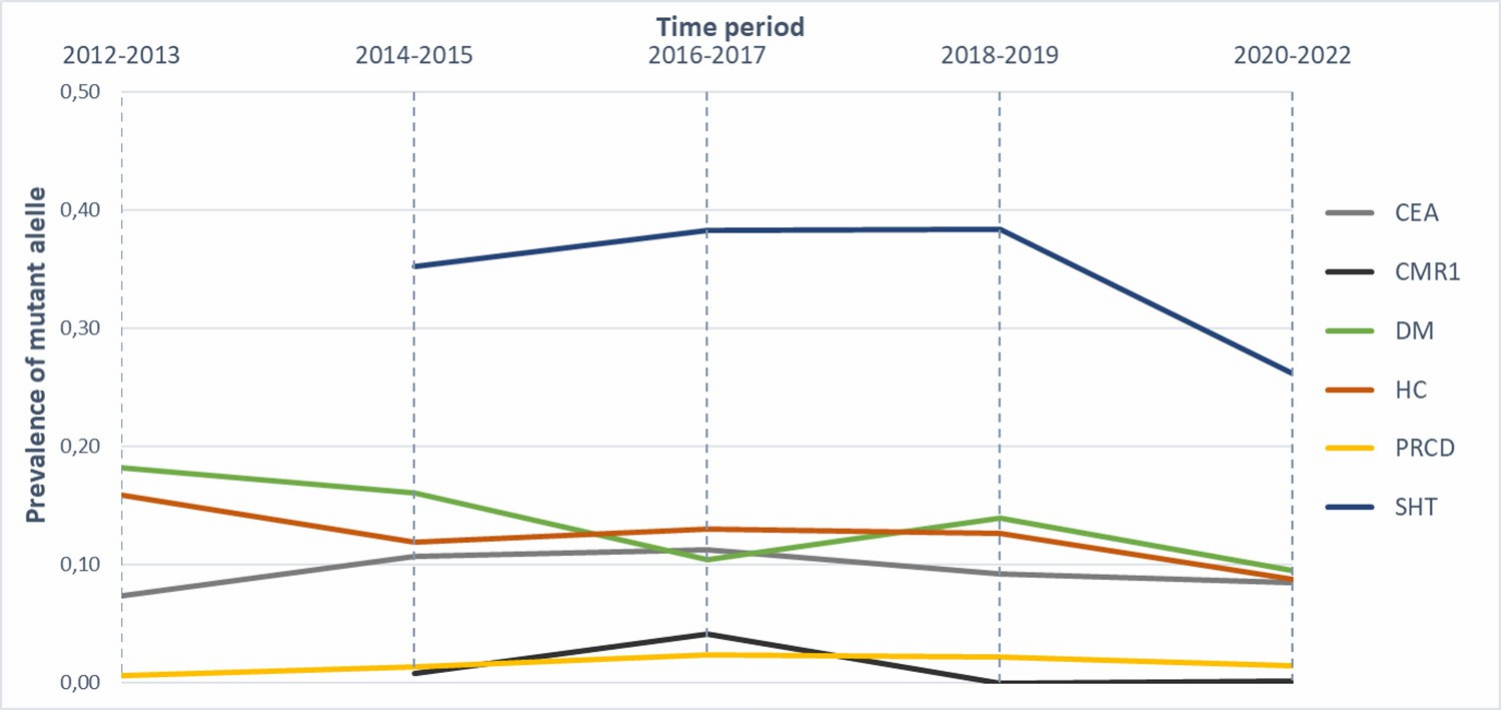
The trends in incidence of the six diseases in the AS breed. The time period: 2012–2022. Each coloured line represents the incidence of the mutant alleles in CEA, CMR1, DM, HC, PRCD and SHT diseases over two-year periods.

**Fig 2:**
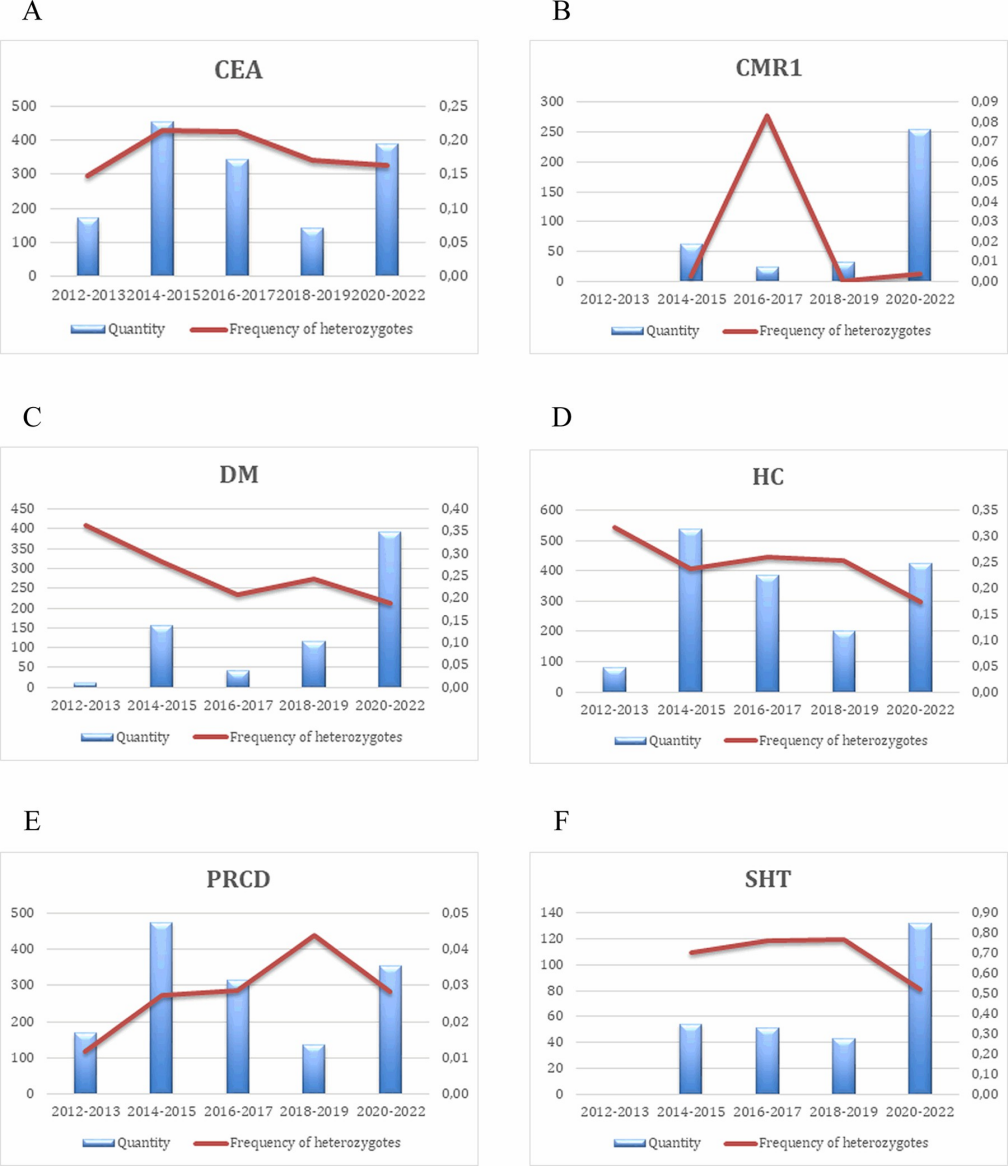
(A-F). The frequencies of the heterozygotes and the number of analysed samples of the six diseases in the AS breed throughout 2012–2022. Blue rectangles—number of samples over the two-year periods, orange line: the frequencies of the heterozygotes.

For six analysed diseases, the highest incidence of the mutant allele was observed for SHT (31.74%), followed by DM (11.77%), HC (11.64%) and CEA (9.71%). For PRA-PRCD and CMR1, the mutant allele was present in low frequency at 1.58% and 0.53%, respectively (Table 2). For each disease, the incidence data are summarised per country in Tables 3–8 (only for the top five countries by the number of samples). The incidence in two-year bins is summarised in Fig 1, and the trends in frequencies of heterozygotes for ten years in two-year bins are summarised in (Fig 2A–2F).

**Table 2:** Summarising the distribution of the mutant and wild-type allelic data of six disorders from all 2595 tested dogs.

Disease	Number of analysed samples	Frequency of mutant allele (%)	Genotype (%)		
			(+/+)	(+/-)	(-/-)
**CEA**	1503	9.71	80.84	18.90	0.26
**CMR1**	376	0.53	98.93	1.06	0
**DM**	722	11.77	77.15	22.16	0.69
**HC**	1641	11.64	76.72	23.28	0
**PRA-PRCD**	1454	1.58	97.04	2.75	0.21
**SHT**	282	31.74	36.52	63.48	0

**Table 3:** Comparison of genotype and mutant allele frequencies in the top five countries for CEA disease.

Country	Number of analysed samples	Frequency of mutant allele (%)	Genotype (%)		
			(+/+)	(+/-)	(-/-)
**France**	557	14.99	75.04	19.93	5.03
**Czech Republic**	226	7.07	85.84	14.16	0
**Germany**	148	7.77	84.46	15.54	0
**Poland**	111	9.46	81.08	18.92	0
**Belgium**	94	5.85	88.3	11.70	0

**Table 4:** Comparison of genotype and mutant allele frequencies in the top five countries for CMR1 disease.

Country	Number of analysed samples	Frequency of mutant allele (%)	Genotype (%)		
			(+/+)	(+/-)	(-/-)
**France**	90	1.11	97.78	2.22	0
**Czech Republic**	86	0.58	98.84	1.16	0
**Austria**	54	0	100	0	0
**Germany**	34	0	100	0	0
**Estonia**	19	0	100	0	0

**Table 5:** Comparison of genotype and mutant allele frequencies in the top five countries for DM disease.

Country	Number of analysed samples	Frequency of mutant allele (%)	Genotype (%)		
			(+/+)	(+/-)	(-/-)
**France**	198	10.1	81.31	17.17	1.52
**Germany**	123	13.41	73.17	26.83	0
**Czech Republic**	105	13.81	73.33	25.72	0.95
**Belgium**	88	11.36	78.41	20.45	1.14
**Austria**	55	10.9	78.18	21.82	0

**Table 6:** Comparison of genotype and mutant allele frequencies in the top five countries for HC disease.

Country	Number of analysed samples	Frequency of mutant allele (%)	Genotype (%)		
			(+/+)	(+/-)	(-/-)
**France**	573	10.21	79.58	20.42	0
**Czech Republic**	287	17.6	64.81	35.19	0
**Germany**	159	3.46	93.08	6.92	0
**Belgium**	149	9.06	81.88	18.12	0
**Austria**	137	8.76	82.48	17.52	0

**Table 7:** Comparison of genotype and mutant allele frequencies in the top five countries for PRA-PRCD disease.

Country	Number of analysed samples	Frequency of mutant allele (%)	Genotype (%)		
			(+/+)	(+/-)	(-/-)
**France**	558	2.15	96.24	3.22	0.54
**Czech Republic**	192	0	100	0	0
**Germany**	174	1.15	97.7	2.3	0
**Austria**	125	4	92	8	0
**Belgium**	118	2.97	94.07	5.93	0

**Table 8:** Comparison of genotype and mutant allele frequencies in the top five countries for SHT disease.

Country	Number of analysed samples	Frequency of mutant allele (%)	Genotype (%)		
			(+/+)	(+/-)	(-/-)
**Austria**	49	20.40	59.18	40.82	0
**Czech Republic**	39	30.77	38.46	61.54	0
**Germany**	35	22.86	54.29	45.71	0
**Poland**	32	43.75	12.5	87.5	0
**France**	31	38.71	22.58	77.42	0

## Discussion

Many canine hereditary diseases and genetic predispositions are recognised and well characterised from clinical signs to the gene defects. Precise diagnostic tools and procedures have been developed to detect the causal mutation responsible for the disease. Breeders should use this information wisely in their breeding program, while preserving the genetic variability of the breed at the same time. Scientists cooperating with cynological organisations and breeders should monitor the incidence of the mutant allele in the population. Unfortunately, although many mutations have already been identified, breeding clubs’ activities in this field are country/club dependent, and that is why such information is almost nonexistent in the complex form. Our goal was to analyse and present such data for AS dog breed in European countries. We have chosen six mutations associated with hereditary diseases in AS. The data for the MDR1 defect was already presented as a part of our other study [4]. It is necessary to note that short-tail is more of a developmental defect than an actual disease, and it is a typical trait for this breed. Nevertheless, in a homozygous state, short-tail mutation leads to embryonic lethality [3].

### Collie eye anomaly (CEA)

In our study, we focused on four ocular disorders (CEA, PRA-PRCD, CMR1, HC), and in this list, CEA was the second most prevalent. The phenotypic development of the disease varies significantly in the CEA-affected animals. A retrospective evaluation of congenital ocular defects in Australia indicated that CEA is the second most common congenital anomaly in AS [11]. In Switzerland, 571 AS were ophthalmologically examined over a period of 8 years (1999–2007), and only one dog was affected with choroidal hypoplasia [34].

The mutant allele frequency in our findings was 9.71%, and almost no homozygous mutants were detected (0.26%), similar to reported results from Italian, Czech and Belgian populations [8, 35, 36]. The slightly lower frequency of the mutant allele observed in these studies was probably caused by their relatively small cohort. The CEA incidence in European countries (Table 3) increased in France in our cohort. The frequency of the mutant allele in the Czech Republic was higher (7.07%) than the one observed by Dostal et al. (4.5%) in 2010 [35].

On the other hand, the CEA frequency reported in Belgium by Beckers et al. (3.1%) was lower than in our survey (5.85%) [36]. We cannot exclude the possibility that it is just the effect of a more significant number of individuals included in our study. Little is known about the incidence of CEA mutation in non-European populations of AS. According to OFA (Orthopaedic Foundation for Animals) statistics based on CEA test results of 128 AS from the North American population, the incidence is 1.98%, which is much lower than in Europe [37]. The frequency of the mutant allele, revealed by the CEA survey in Thailand, was 5.1% [38]. It could be an effect of genetic drift, but further large-scale studies are required to confirm such a hypothesis.

### Canine multifocal retinopathy (CMR)

CMR belongs to relatively rare ocular defects. For the presence of *cmr1* mutation, 376 individuals were analysed. Of all investigated defects, *cmr1* mutant allele occurred at the lowest frequency, 0.53% and was found only in the heterozygous state. It was approximately five times lower (2.85%) than reported in an extensive survey which also included 140 AS [39], but higher than in OFA testing statistics, where in the cohort of 74 individuals, the mutant allele was not detected [37]. A closer look at the mutation frequency in European countries with a sufficient number of individuals showed a moderate frequency increase in the AS population in France (Table 4).

### Degenerative myelopathy (DM)

The mutation is widespread in many dog breeds, including AS. DM is the only disease in this study where we observed a relatively increased frequency of dogs homozygous for the mutant allele. Zeng et al. examined 113 AS individuals, 31.9% were homozygous affected, and 17.7% were heterozygotes for the mutation [40]. In the OFA statistics covering 276 dogs from the North American population of AS, carriers represented 19.9% and homozygous affected 13.4% [37]. The mutant allele frequencies were 40.75% and 23.35%, respectively. In our findings, only 0.69% were homozygous for the mutant allele, and 22.16% were heterozygotes. The incidence of the mutant allele was at least two times lower than in the American population. The different frequencies observed may be due to the different sizes of analysed populations or, more probably, because these populations are geographically separated. It could be related to genetic drift during the import of AS to Europe. There are only minor differences in the incidence of *sod1* mutation in European countries (Table 5).

### Hereditary cataract (HC)

In the present study, carriers represented 23.28% of the AS population, and the remaining 76.72% were homozygous healthy dogs. The incidence of *hsf4* mutant allele was the highest of all hereditary ocular diseases in our study. In 2009, Mellersh et al. investigated 392 individuals with comparable results to ours (HC +/+ 70.6%, HC +/- 25.6% and HC -/- 3.8%) [23]. Mellers et al. reported a slightly higher percentage of the mutant allele (16.6% vs 11.64%), but it should be noted that their cohort included a group of dogs with clinically verified cataracts [23]. In contrast, two other studies observed a lower frequency of the mutant allele (6.95% and 7.8%), but their cohorts were much smaller (140 and 32 individuals) [36, 39]. Among European countries, the highest frequency was observed in the Czech Republic (17.6%) and the lowest in Germany (3.46%) (Table 6). We can only speculate if this observation is related to different breeding rules in these countries or is just a coincidence in analysed groups.

### Progressive rod-cone degeneration (PRCD)

PRCD is an inherited disease that occurs in many dog breeds and manifests in various forms. The mutation frequency was low in the tested population for this inherited disease (1.58%). Moreover, less than 3% of the analysed dogs were heterozygotes, which led us to conclude that PRCD appears rare in the AS breed. It is further supported by the OFA report, with an incidence of 1.22% [37]. The sequence was slightly increased in Austria and Belgium (Table 7). In other breeds, for example, English Cocker Spaniels, the high frequency of mutant allele was observed (25.5%) [28].

### Short tail (SHT)

According to our observations, a high percentage (63.48%) of heterozygotes was found, and the highest frequency of the mutant allele was observed in comparison to other diseases (Table 2). A short tail is caused by an autosomal dominant mutation in the T-box of the transcription factor T gene [32]. Individuals with short tails carry the mutation in a heterozygous state. The mutation in a homozygous state has fatal consequences and leads to death. Thus, mating two heterozygotes is not recommended. The mating of two heterozygous individuals showed a 30% loss of puppies per litter [3, 32]. Currently, due to legislation, tail docking is restricted in many countries. Since the traditional look is important for many owners, breeders may tend to produce more natural “bobtails”. We are also aware that some of the results may be biased as they come from routine diagnostics, and dogs with standard tail lengths are not tested for the presence of the mutation, which may overestimate the mutation frequency.

In addition to the overall incidence of analysed diseases, we have also investigated how disease allele incidence and carrier frequencies have developed over time (Figs 1 and 2). We have observed a moderate decrease in incidence, but except SHT, all diseases were present in relatively low frequencies. This observation concurs with a large-scale survey, which determined changes in the frequency of disease-causing mutations after introducing a commercial DNA test [41]. They found that data from test results show a slight general decline in either the mutation frequency or the proportion of carriers.

Since its official registration as a purebred breed, AS, like the other modern breeds, had to face common breeding practices, which amplified the incidence of autosomal recessive genetic disorders. The complex data about the incidence of known hereditary diseases in the European population are almost non-existent; therefore, our aim was to reveal the actual state. The highest incidence was observed for SHT mutation, which was not surprising, since short-tail is a typical trait for AS, although a homozygous state leads to embryonal death. For three other diseases (HC, DM, CEA), the incidence of the mutant allele was approximately 10%, and they require breeders’ attention when planning to mate. Moreover, HC disorder in AS has an autosomal dominant mode of inheritance with incomplete penetrance. Therefore, use of heterozygotes should be carefully considered in breeding. The remaining two diseases (PRCD, CMR1) showed a relatively low frequency of the mutant allele and represented less risky diseases, but due to the nature of modern breeding, their incidence should be monitored.

## Conclusion

This study gives an overview of six hereditary diseases frequently present in the AS breed and the mutant alleles’ incidence for each of them. Altogether, we tested 2595 AS individuals during a 10-year time span. The findings of this study suggest controlling the mating process by testing all mating individuals and choosing, if possible, clinically healthy dogs. Healthy carriers should be kept in breeding programs if the selected mate is homozygous clear. Any eradication of disease alleles should happen slowly and gradually so that loss of genetic diversity is avoided as much as possible. This controlled elimination can significantly reduce affected alleles in the population and emphasise the importance of genotyping as a method of early diagnosis.

## Supporting information

S1 Table Primers used for amplifications of PCR products for CEA, CMR1, DM, HC, PRA-PRCD and ST. (DOCX)

## References

[1] Alderton D. Encyclopedia of Dogs. Parragon; 2008.

[2] The Australian Shepherd [Internet]. ASCA. 2022 [cited 2022 Oct 24]. Available from: https://asca.org/aussies/about-aussies/the-australian-shepherd/

[3] Indrebø A, Langeland M, Juul HM, Skogmo HK, Rengmark AH, Lingaas F. A study of inherited short tail and taillessness in Pembroke Welsh corgi. J Small Anim Pract. 2008 May;49(5):220–4. doi: 10.1111/j.1748-5827.2007.00435.x 17850278

[4] Firdova Z, Turnova E, Bielikova M, Turna J, Dudas A. The prevalence of ABCB1:c.227_230delATAG mutation in affected dog breeds from European countries. Res Vet Sci. 2016 Jun;106:89–92. doi: 10.1016/j.rvsc.2016.03.016 27234542

[5] Parker HG, Kukekova AV, Akey DT, Goldstein O, Kirkness EF, Baysac KC, et al. Breed relationships facilitate fine-mapping studies: a 7.8-kb deletion cosegregates with Collie eye anomaly across multiple dog breeds. Genome Res. 2007 Nov;17(11):1562–71. doi: 10.1101/gr.6772807 17916641 PMC2045139

[6] Chang H-S, Mizukami K, Yabuki A, Hossain MA, Rahman MM, Uddin MM, et al. A novel rapid genotyping technique for Collie eye anomaly: SYBR Green-based real-time polymerase chain reaction method applicable to blood and saliva specimens on Flinders Technology Associates filter paper. J Vet Diagn Invest. 2010 Sep;22(5):708–15. doi: 10.1177/104063871002200506 20807925

[7] Mizukami K, Chang H-S, Ota M, Yabuki A, Hossain MA, Rahman MM, et al. Collie eye anomaly in Hokkaido dogs: case study. Vet Ophthalmol. 2012 Mar;15(2):128–32. doi: 10.1111/j.1463-5224.2011.00950.x 22051190

[8] Marelli SP, Rizzi R, Paganelli A, Bagardi M, Minozzi G, Brambilla PG, et al. Genotypic and allelic frequency of a mutation in the NHEJ1 gene associated with collie eye anomaly in dogs in Italy. Vet Rec Open. 2022 Dec;9(1):e26. doi: 10.1002/vro2.26 35127102 PMC8800487

[9] Lowe JK, Kukekova AV, Kirkness EF, Langlois MC, Aguirre GD, Acland GM, et al. Linkage mapping of the primary disease locus for collie eye anomaly. Genomics. 2003 Jul;82(1):86–95. doi: 10.1016/s0888-7543(03)00078-8 12809679

[10] Kucharczyk N, Cislo-Pakuluk A, Bedford P. Collie Eye Anomaly in Australian Kelpie dogs in Poland. BMC Vet Res. 2019 Nov 4;15(1):392. doi: 10.1186/s12917-019-2143-y 31684941 PMC6829813

[11] Munyard KA, Sherry CR, Sherry L. A retrospective evaluation of congenital ocular defects in Australian Shepherd dogs in Australia. Vet Ophthalmol. 2007 Jan;10(1):19–22. doi: 10.1111/j.1463-5224.2007.00486.x 17204124

[12] Gornik KR, Pirie CG, Duker JS, Boudrieau RJ. Canine multifocal retinopathy caused by a BEST1 mutation in a Boerboel. Vet Ophthalmol. 2014 Sep;17(5):368–72. doi: 10.1111/vop.12095 23998685

[13] Hoffmann I, Guziewicz KE, Zangerl B, Aguirre GD, Mardin CY. Canine multifocal retinopathy in the Australian Shepherd: a case report. Vet Ophthalmol. 2012 Sep;15 Suppl 2:134–8. doi: 10.1111/j.1463-5224.2012.01005.x 22432598 PMC3787078

[14] Miyadera K, Acland GM, Aguirre GD. Genetic and phenotypic variations of inherited retinal diseases in dogs: the power of within- and across-breed studies. Mamm Genome. 2012 Feb;23(1–2):40–61. doi: 10.1007/s00335-011-9361-3 22065099 PMC3942498

[15] Johnson AA, Guziewicz KE, Lee CJ, Kalathur RC, Pulido JS, Marmorstein LY, et al. Bestrophin 1 and retinal disease. Prog Retin Eye Res. 2017 May;58:45–69. doi: 10.1016/j.preteyeres.2017.01.006 28153808 PMC5600499

[16] Guziewicz KE, Zangerl B, Lindauer SJ, Mullins RF, Sandmeyer LS, Grahn BH, et al. Bestrophin gene mutations cause canine multifocal retinopathy: a novel animal model for best disease. Invest Ophthalmol Vis Sci. 2007 May;48(5):1959–67. doi: 10.1167/iovs.06-1374 17460247 PMC1931491

[17] Nardone R, Höller Y, Taylor AC, Lochner P, Tezzon F, Golaszewski S, et al. Canine degenerative myelopathy: a model of human amyotrophic lateral sclerosis. Zoology. 2016 Feb;119(1):64–73. doi: 10.1016/j.zool.2015.09.003 26432396

[18] Awano T, Johnson GS, Wade CM, Katz ML, Johnson GC, Taylor JF, et al. Genome-wide association analysis reveals a SOD1 mutation in canine degenerative myelopathy that resembles amyotrophic lateral sclerosis. Proc Natl Acad Sci U S A. 2009 Feb 24;106(8):2794–9. doi: 10.1073/pnas.0812297106 19188595 PMC2634802

[19] Kato S. Amyotrophic lateral sclerosis models and human neuropathology: similarities and differences. Acta Neuropathol. 2008 Jan;115(1):97–114. doi: 10.1007/s00401-007-0308-4 18026741

[20] Nakamae S, Kobatake Y, Suzuki R, Tsukui T, Kato S, Yamato O, et al. Accumulation and aggregate formation of mutant superoxide dismutase 1 in canine degenerative myelopathy. Neuroscience. 2015 Sep 10;303:229–40. doi: 10.1016/j.neuroscience.2015.06.066 26162235

[21] Mellersh CS, Graves KT, McLaughlin B, Ennis RB, Pettitt L, Vaudin M, et al. Mutation in HSF4 associated with early but not late-onset hereditary cataract in the Boston Terrier. J Hered. 2007 Jul 4;98(5):531–3. doi: 10.1093/jhered/esm043 17611257

[22] Mellersh CS, Pettitt L, Forman OP, Vaudin M, Barnett KC. Identification of mutations in HSF4 in dogs of three different breeds with hereditary cataracts. Vet Ophthalmol. 2006 Sep;9(5):369–78. doi: 10.1111/j.1463-5224.2006.00496.x 16939467

[23] Mellersh CS, McLaughlin B, Ahonen S, Pettitt L, Lohi H, Barnett KC. Mutation in HSF4 is associated with hereditary cataract in the Australian Shepherd. Vet Ophthalmol. 2009 Nov;12(6):372–8. doi: 10.1111/j.1463-5224.2009.00735.x 19883468

[24] Engelhardt A, Wöhlke A, Distl O. Evaluation of canine heat-shock transcription factor 4 as a candidate for primary cataracts in English Cocker Spaniels and wire-haired Kromfohrlanders. J Anim Breed Genet. 2007 Aug;124(4):242–5. doi: 10.1111/j.1439-0388.2007.00663.x 17651328

[25] Zangerl B, Goldstein O, Philp AR, Lindauer SJP, Pearce-Kelling SE, Mullins RF, et al. Identical mutation in a novel retinal gene causes progressive rod-cone degeneration in dogs and retinitis pigmentosa in humans. Genomics. 2006 Nov;88(5):551–63. doi: 10.1016/j.ygeno.2006.07.007 16938425 PMC3989879

[26] Acland GM, Ray K, Mellersh CS, Gu W, Langston AA, Rine J, et al. Linkage analysis and comparative mapping of canine progressive rod-cone degeneration (prcd) establishes potential locus homology with retinitis pigmentosa (RP17) in humans. Proc Natl Acad Sci U S A. 1998 Mar 17;95(6):3048–53. doi: 10.1073/pnas.95.6.3048 9501213 PMC19692

[27] Remez L, Zobor D, Kohl S, Ben-Yosef T. The progressive rod-cone degeneration (PRCD) protein is secreted through the conventional ER/Golgi-dependent pathway. Exp Eye Res. 2014 Aug;125:217–25. doi: 10.1016/j.exer.2014.06.017 24992209

[28] Andrade LR, Caceres AM, Trecenti AS, Brandão CVS, Gandolfi MG, Aguiar EV, et al. Allele Frequency of the C.5G>A Mutation in the PRCD Gene Responsible for Progressive Retinal Atrophy in English Cocker Spaniel Dogs. Animals (Basel) [Internet]. 2019 Oct 21;9(10). Available from: 10.3390/ani9100844 PMC6826964 31640229

[29] Spencer WJ, Arshavsky VY. PRCD Is a Small Disc-Specific Rhodopsin-Binding Protein of Unknown Function. Adv Exp Med Biol. 2019;1185:531–5. doi: 10.1007/978-3-030-27378-1_87 31884666

[30] Spencer WJ, Ding J-D, Lewis TR, Yu C, Phan S, Pearring JN, et al. PRCD is essential for high-fidelity photoreceptor disc formation. Proc Natl Acad Sci U S A. 2019 Jun 25;116(26):13087–96. doi: 10.1073/pnas.1906421116 31189593 PMC6601265

[31] Murgiano L, Becker D, Spector C, Carlin K, Santana E, Niggel JK, et al. CCDC66 frameshift variant associated with a new form of early-onset progressive retinal atrophy in Portuguese Water Dogs. Sci Rep. 2020 Dec 3;10(1):21162. doi: 10.1038/s41598-020-77980-5 33273526 PMC7712861

[32] Haworth K, Putt W, Cattanach B, Breen M, Binns M, Lingaas F, et al. Canine homolog of the T-box transcription factor T; failure of the protein to bind to its DNA target leads to a short-tail phenotype. Mamm Genome. 2001 Mar;12(3):212–8. doi: 10.1007/s003350010253 11252170

[33] Hytönen MK, Grall A, Hédan B, Dréano S, Seguin SJ, Delattre D, et al. Ancestral T-box mutation is present in many, but not all, short-tailed dog breeds. J Hered. 2009 Mar;100(2):236–40. doi: 10.1093/jhered/esn085 18854372

[34] Walser-Reinhardt L, Hässig M, Spiess B. Collie Eye Anomaly in Switzerland. Schweiz Arch Tierheilkd. 2009 Dec;151(12):597–603. doi: 10.1024/0036-7281.151.12.597 19946851

[35] Dostál J, Horák P, Hrdlicová A, Stratil A. Simplified PCR analysis of a mutation in the NHEJ1 gene causing collie eye anomaly in some dog breeds. Czech J Anim Sci. 2010 Aug 19;55(8):346–50.

[36] Beckers E, Van Poucke M, Ronsyn L, Peelman L. Frequency estimation of disease-causing mutations in the Belgian population of some dog breeds, part 1: shepherds. Vlaams Diergeneeskd Tijdschr. 2016;85(4):175–84.

[37] Browse By Breed [Internet]. OFA. 2021 [cited 2022 Oct 24]. Available from: https://ofa.org/chic-programs/browse-by-breed/?breed=AS

[38] Lerdkrai C, Phungphosop N. A novel multiplex polymerase chain reaction assay for the genotypic survey of the non-homologous end-joining factor 1 gene associated with Collie eye anomaly in Thailand. Vet World. 2022 Jan;15(1):132–9. doi: 10.14202/vetworld.2022.132-139 35369581 PMC8924400

[39] Donner J, Kaukonen M, Anderson H, Möller F, Kyöstilä K, Sankari S, et al. Genetic Panel Screening of Nearly 100 Mutations Reveals New Insights into the Breed Distribution of Risk Variants for Canine Hereditary Disorders. PLoS One. 2016 Aug 15;11(8):e0161005. doi: 10.1371/journal.pone.0161005 27525650 PMC4985128

[40] Zeng R, Coates JR, Johnson GC, Hansen L, Awano T, Kolicheski A, et al. Breed distribution of SOD1 alleles previously associated with canine degenerative myelopathy. J Vet Intern Med. 2014;28(2):515–21. doi: 10.1111/jvim.12317 24524809 PMC4238831

[41] Lewis TW, Mellersh CS. Changes in mutation frequency of eight Mendelian inherited disorders in eight pedigree dog populations following introduction of a commercial DNA test. PLoS One. 2019 Jan 16;14(1):e0209864. doi: 10.1371/journal.pone.0209864 30650096 PMC6334900

