# Supplementary material for: The incidence of genetic disease alleles in Australian Shepherd dog breed in European countries
Source: PLoS One. 2023 Feb 27;18(2):e0281215. doi: 10.1371/journal.pone.0281215 (PMC9970066; doi:10.1371/journal.pone.0281215)
Supplement: S1 Table — (DOCX) [file pone.0281215.s001.docx]

**S1 Table. Primers used for amplifications of PCR products for CEA, CMR1, DM, HC, PRA-PRCD and ST**

| **Disease** | **Primer name** | **Affected gene** | **Sequence of primer 5’-3’** | **Product length** | **Reference** |
| --- | --- | --- | --- | --- | --- |
| **Collie eye anomaly** | CEA1 F | NHEJ1 | tctcacaggcagaaagctca | 610 | [5] |
|  | CEA1 R |  | ccattcattcctttgccagt |  |  |
|  | CEA2 F |  | tgggctggtgaacatttgta | 870 |  |
|  | CEA2 R |  | cctttttgtttgccctcaga |  |  |
| **Canine multifocal retinopathy, type 1** | CMR1 F | BEST1 VDM2 | gaccgtcacctactcaagccaag | 257 | [16] |
|  | CMR1 R |  | cagccttacaggtctctggtacatc |  |  |
| **Degenerative myelopathy** | DM2 F | SOD1 | gtgggcctgttgtggtatca | 75 | [18] |
|  | DM2 R |  | caaactgatggacgtggaatcc |  |  |
| **Primary hereditary cataract** | HSF4 F2 | HSF4 | ggtctggcagcctagatggc | 549 | [22] |
|  | HSF4 R |  | ttccaataccgaaatgaactcctac |  |  |
| **Progressive rod-cone degeneration** | PRCD F | PRCD | ccagtggcagcaggaacc | 512 | [25] |
|  | PRCD R |  | ccgacctgctgcccacgactg |  |  |
| **Short tail** | SHT F | T - gene | cgagtggaccacctgctgag | 384 | [33] |
|  | SHT R |  | gccgagcagaaaggagcaagaag |  |  |
